# Helping in humans and other animals: a fruitful interdisciplinary dialogue

**DOI:** 10.1098/rspb.2017.0929

**Published:** 2017-09-27

**Authors:** Redouan Bshary, Nichola J. Raihani

**Affiliations:** 1 Institute of Biology, University of Neuchâtel, Emile-Argand 11, 2000 Neuchâtel, Switzerland; 2 Department of Experimental Psychology, University College London, 26 Bedford Way, London WC1H 0AP, UK

**Keywords:** helping, cooperation, altruism, comparative approach, humans, game theory

## Abstract

Humans are arguably unique in the extent and scale of cooperation with unrelated individuals. While pairwise interactions among non-relatives occur in some non-human species, there is scant evidence of the large-scale, often unconditional prosociality that characterizes human social behaviour. Consequently, one may ask whether research on cooperation in humans can offer general insights to researchers working on similar questions in non-human species, and whether research on humans should be published in biology journals. We contend that the answer to both of these questions is yes. Most importantly, social behaviour in humans and other species operates under the same evolutionary framework. Moreover, we highlight how an open dialogue between different fields can inspire studies on humans and non-human species, leading to novel approaches and insights. Biology journals should encourage these discussions rather than drawing artificial boundaries between disciplines. Shared current and future challenges are to study helping in ecologically relevant contexts in order to correctly interpret how payoff matrices translate into inclusive fitness, and to integrate mechanisms into the hitherto largely functional theory. We can and should study human cooperation within a comparative framework in order to gain a full understanding of the evolution of helping.

## 1. Introduction

Helping behaviours that increase the direct fitness of recipients underpin several major evolutionary transitions [1]. Acts in which helpers provide any resource (e.g. food, time) are interesting because evolutionary theory strongly emphasizes the importance of competition and selfish behaviour. Humans are adept at helping each other. From a quantitative perspective, this trait is not unique in the animal kingdom; arguably, hymenopterans and other eusocial species are even more helpful within their colonies. However, helping by the latter is explained by biological altruism based on kin selection [2,3], while humans also cooperate with unrelated individuals for direct fitness benefits on a scale that is unmatched by any other species. Importantly, the criteria for cooperating are highly flexible: the same individual may cooperate with friends, colleagues, supporters of the same football club, political affiliates, compatriots or even international alliances. Help can be provided in different currencies (e.g. time/money/physical effort) and is also often provided in situations where it is unclear how return benefits may be accrued, from letting a car out at a busy junction to donating to victims of natural disasters in far-away countries.

The frequency and scale of human helping could depend on several factors that appear to be unique to humans: our capacity for spoken and written language, the use of tags to identify groups, societal-level norms and institutions that both prescribe cooperation and punish defection, various media channels that allow for large-scale communication and coordination, and banks to transfer money—a unique non-perishable resource—anywhere. One might therefore wonder to what extent research on human cooperation yields idiosyncratic explanations, rendering comparisons with other species useless. One may also ask whether research on human cooperation is suitable for publication in biological journals like *Proceedings of the Royal Society B*. Here, we address this question. We first summarize briefly the enormous impact that theoretical concepts and empirical studies of human cooperation have had on research in other species. We then highlight topics of interdisciplinary interest and shared future challenges. It should become clear that we favour an open-minded and inclusive approach, where humans are just another species that can be studied under the general framework of evolutionary theory. While human cooperation might be more peculiar than cooperation in many non-human species, each species would appear unique if every detail was taken into consideration. Therefore, a distinction between disciplines based on study organisms only hinders progress.

## 2. Theory on human helping as inspiration for biological research

**Figure 1.**
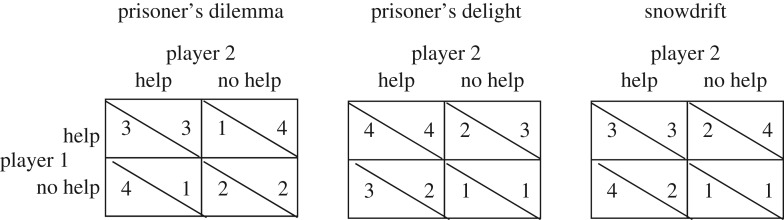
Three stylized economic games that differ with respect to the payoff matrix. In the prisoner's dilemma, not helping yields a higher payoff in each interaction no matter how the partner behaves, which makes helping an investment that needs to yield future benefits. Thus, iterated interactions are required for conditional helping to evolve. In the prisoner's delight, helping yields a higher payoff no matter how the partner behaves, which makes helping a self-serving action, even in a single-round game. In the snowdrift game, the best choice depends on the partner's action: help if the partner does not help and do not help if the partner helps. Under these circumstances helping is under negative frequency-dependent selection in a single-round game.

Theoretical approaches to understand helping in humans predate evolutionary concepts of helping. The principal tools used by biologists were developed by economists in the form of game theory—a framework to understand how humans should make decisions in strategic interactions [4]. A ‘game’ is a formal mathematical model of an interaction, defining the payoffs to all players. A key insight is that players' payoffs are affected by their own decisions and also by those of their partner(s). Thus, the dominant strategy depends on the strategy that is used by the partner(s). Economists assume that payoffs translate into utility and that players maximize utility. Stylized economic games were developed to study optimal decision rules. In their simplest form, these games consist of two players who can each choose between two actions, for example to cooperate or to defect. Games can be one-shot or repeated over a number of rounds. The resulting payoffs of action combinations can be captured by a 2 × 2 matrix. The matrices for well-known games [5], like the prisoner's dilemma game, the prisoner's delight game and the snowdrift game (also called hawk–dove game) are summarized in figure 1. These games were subsequently adopted by evolutionary biologists to explore when helping behaviour could be evolutionarily stable [6] in a population. Under this evolutionary approach, strategies are inherited traits that specify behaviours [7]. Rather than utility, evolutionary biologists assume that payoffs translate into fitness, with the accompanying assumption that strategies which, on average, increase fitness will be under positive selection.

A common goal is to understand why individuals should provide help to others. Economists, never considering the genetic structure of human populations, focused on how helping may increase on average the direct fitness of that actor. This form of helping has been termed ‘mutual benefits’ [8] or ‘cooperation’ [9]. We will use the latter term in this paper, and restrict the term ‘mutualism’ to describe mutual helping between species [10]. Economists demonstrated that cooperative solutions are possible, when the number of rounds times the benefits of mutual cooperation outweigh the cost of cooperating (folk theorem [5,11]). Evolutionary theorists subsequently rediscovered this principle verbally [12] and then mathematically, albeit with limited generality [13]. Economists also showed how supply and demand determine exchange rates [14], an insight that was then incorporated into biological market theory [15,16]. Similarly, the idea that reputational effects in a communication network may affect animal behaviour [17] was foreshadowed by concepts explicitly developed by economists to understand human cooperation [18,19].

Evolutionary biology provided a major conceptual insight thanks to Hamilton's kin selection theory [2,3]. Helping may be altruistic in biological terms, by reducing the actor's lifetime reproductive success, and yet still be positively selected if helper and recipient are related (specifically, when *rB* − *C* > 0, where *r* = relatedness between actor and beneficiary, *B* = fitness benefit conferred on beneficiary and *C* = personal fitness cost incurred by actor [2,3]). Thus, the one-sided borrowing from biologists eventually became a fruitful dialogue, not least because cooperation and biological altruism may act simultaneously to promote selection on helping, including in humans (e.g. [20–22]). Indeed, game-theoretic approaches have become increasingly prominent in the attempt to understand the evolution of helping behaviour [7,23]. Importantly, the logic underpinning game-theoretical models of behaviour reflects general principles in evolutionary theory and may hence be applied to any species, including humans.

## 3. Empirical research on human helping as inspiration for animal research

We focus on supposed examples of cooperation based on investments. We define an investment as a behaviour that reduces the current payoff of the actor and increases the current payoff of the recipient. Cooperation based on investment appears to be vulnerable to cheaters who do not invest but receive investment from others. A vast theoretical literature has shown that higher-level selection processes (kin/group selection, interdependencies between individuals) may select against cheating. These processes have been relatively neglected in empirical studies, partly because of the difficulty of quantifying them. More ecologically motivated future research may hence reveal that some apparent investments are actually self-serving forms of helping [24]. As with theoretical concepts, empirical research on human helping has had a serious head start over similar research on non-human animals. It is impossible to summarize the existing literature on human helping adequately here. Though most of this research focuses on understanding human social behaviour only, the data and conclusions nevertheless provide inspiration to researchers studying non-human animals, who might look for similar behaviours in their own study systems.

In this context, it is important to distinguish ultimate from proximate questions [25]. Ultimate questions address the adaptive value of helping, which is rather simple: helpful strategies can only be under positive selection if they provide lifetime fitness benefits (+/+) to all participants, the exception being biological altruism (−/+) based on kin selection. Thus, from an ultimate perspective, there is no *a priori* reason to demarcate research aimed at understanding the evolution of costly social behaviour in humans from similar research on other species. In contrast, the proximate mechanisms underlying social decision-making can be highly diverse: genetic predispositions, physiological states and cognitive mechanisms may all interact to produce social behaviour, and humans might often use idiosyncratic proximate mechanisms to achieve cooperation. Examples of these include mentalizing, fairness preferences, cultural norms, shared intentionality, and the ability to communicate intentions using gestures (such as pointing) and language. These abilities may not be unique to humans, but they are unarguably more pronounced in humans than in any other species. Moreover, variation in proximate mechanisms can affect the means by which cooperation is achieved—and sometimes even the possibility to achieve it [26]. We therefore discuss research on ultimate and proximate explanations for costly social strategies separately.

### (a) Ultimate explanations

Humans appear to be an excellent model species to test the predictions of evolutionary game theory. Experimenters can construct precise material payoffs for any possible combination of individual decisions, decide how many rounds are played with whom, and how much information subjects obtain. To understand the adaptive significance of costly helping behaviour, many studies on humans have identified partner control mechanisms—responses to being cheated that reduce the cheater's payoff [27]. These include tit-for-tat-like reciprocity, punishment, reputation effects, partner choice and (relatedly) ostracism (e.g. [28–31]).

After an initial focus on tit-for-tat-like reciprocity (reviewed in [32]), biologists also searched for examples of these same partner control mechanisms in non-human species. Marine cleaning mutualism involving the cleaner wrasse *Labroides dimidiatus* provided experimental support for all these control mechanisms. Cleaners remove ectoparasites from visiting ‘client’ reef fishes [33]. Nevertheless, conflict arises because cleaners prefer to eat client mucus, which constitutes cheating. Therefore, clients have to make cleaners feed against their preference to receive a good service [34]. Partner control mechanisms become visible when clients respond to cleaners taking a bite of mucus (which correlates with clients visibly jolting in response to cleaner mouth contact). As summarized in [34], client species with access to a single cleaning station punish cleaners through aggressive chases, while clients with access to several cleaning stations terminate the interaction and visit another cleaner for their next inspection. In addition, clients arriving at a cleaning station extract information from any ongoing interaction and invite for inspection only if the cleaner behaves cooperatively. Thus, the cleaner's reputation depends on their behaviour, and they behave more cooperatively if they are observed. Finally, the larger cleaner males may also punish their female partner for cheating a jointly inspected client, a simple form of third party punishment [35] that is fine-tuned to the stakes (i.e. the quality of the client as a food source) [36].

Research on cleaning mutualism was partly inspired by classic studies on the effects of punishment and reputation on human cooperation, which highlighted that the possibility of being punished or being chosen for interactions by observers, respectively, could both promote cooperation at higher levels than when these incentives were absent (e.g. [28,30]). Meanwhile, results from the cleaner fish mutualism have, in turn, inspired subsequent studies on partner choice and asymmetric punishment in humans, for example leading us to investigate whether punishment or partner choice is a more effective incentive to cooperate when both incentives are co-present [37], and to explore whether power asymmetries increase the efficacy of punishment as a cooperation-enforcing mechanism in two-player games [38].

**Figure 2.**
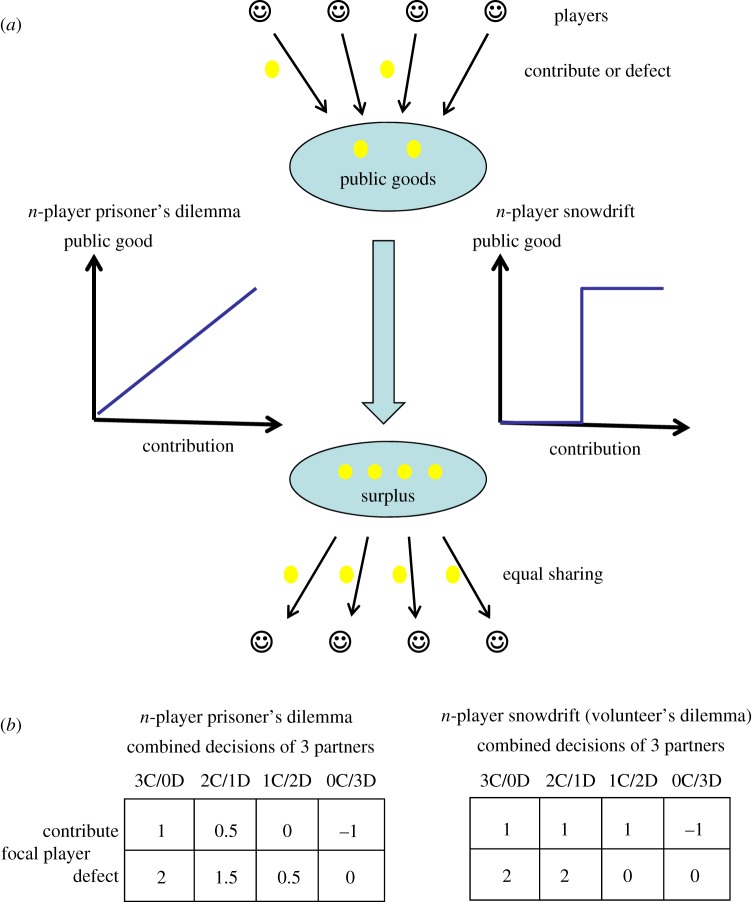
Public goods games. (*a*) Contribution to a public good creates a surplus. In an *n*-player prisoner's dilemma game the created value is a linear function of the amount contributed, while in a *n*-player snowdrift game it is nonlinear (a step function in the figure). The created value is then shared equally among players irrespective of initial contributions. (*b*) Case examples for the payoffs of a focal player depending on whether she contributes or defects and what her three partners are doing. In the *n*-player prisoner's dilemma, it is assumed that contributing costs 1 unit and generates a value of 2 units. In the *n*-player snowdrift it is assumed that contributing costs 1 unit and that 2 contributions are needed to produce a public good of 8 units.

Unlike most non-human species, humans regularly cooperate in large groups of unrelated individuals. Economists and social scientists have therefore pioneered the study of cooperation in groups. The payoffs can be captured using public goods games, where benefits are assumed to be either a linear or a sigmoid function of investments (figure 2). Under the former assumption, the interaction is an *n*-player prisoner's dilemma and investments therefore risk being biologically altruistic. Where benefits are a nonlinear function of investments, then the interaction is an *n*-player snowdrift game (a volunteer's dilemma) and contributions are negatively frequency dependent (figure 2) [39,40]. Again, claims about human uniqueness with respect to *n*-player cooperation have inspired biologists interested in a comparative approach to find suitable non-human model systems in which to apply the human literature on public goods games. Importantly, the most suitable species will not necessarily be the species that are phylogenetically most related to humans, but those that routinely interact in *n*-player social dilemmas (with non-relatives)—such that *n*-player social dilemmas constitute an ecologically valid scenario. To this end, species that regularly engage in inter-group conflict may provide a promising arena. Humans promote cooperation in larger groups by providing incentives: rewarding contributors to the public good and punishing so-called free-riders [28,41]. Similarly, female vervet monkeys use these same incentives to increase male participation in inter-group conflicts [42].

One of the key difficulties in identifying *n*-player public goods games outside of humans is to obtain informed estimates of both the precise payoff matrices and the fitness consequences. In some cases, in contrast to the frameworks described above, individual actions appear to be self-serving and to provide public goods only as a by-product (e.g. punishment of scale-eating sabre-tooth blennies by their victims [43]; group hunting of multiple non-shared prey [44]). In contrast, many terrestrial group hunting examples involve the killing of a single large prey, where individual payoffs depend crucially on how the prey is shared rather than on the increased hunting success [45]. In such cases, payoffs are affected by ownership, contribution to the hunt, sex and/or position in the hierarchy (e.g. [46–49]), variables that are not typically considered in standard public goods games (but see [50]). Many examples of *n*-player public goods have been described in microbes, where the production of extracellular molecules constitutes an investment that can provide benefits to non-producers (reviewed in [51]). Since increased production typically yields diminishing benefits, many of these examples yield fitness consequences that correspond to the volunteer's dilemma payoff matrix [40]. These various case studies highlight an important issue: despite the continued focus on *n*-player prisoner's dilemma payoffs in human laboratory studies, many public goods in humans might also better approximate the nonlinear payoffs of snowdrift/volunteer's dilemma games [40,52]. A key priority for future research on humans is therefore to evaluate the payoffs of real-world interactions and design experiments to capture these in the laboratory.

### (b) Proximate explanations

Research on the cognitive mechanisms underpinning human helping might initially appear of little value for understanding helping in other species. This is because humans have a cognitive toolbox that is unmatched by any other species (though there is considerable debate regarding the extent to which differences are qualitative or only quantitative [53]). Many of these cognitive tools are tightly linked to/enhanced by human language, which is in itself arguably the most important tool. Spoken and written language does not only allow for basic communication about behaviour; it also facilitates negotiation, coordination, the expression of some emotions and the establishment of shared intentionality. Language is also the basis for some forms of teaching [54] and the establishment of shared cultural norms. Culture in turn provides a variety of cues that can be used to generate cooperation even between strangers. It seems highly likely that there is a tight link between our cognitive abilities and our ability to cooperate, though it remains unclear whether ecological pressures to cooperate selected for our cognitive abilities or whether these abilities created opportunities for extreme cooperation. Comparative research that evaluates what cognitive processes are used by humans and other species during social interactions might help address this question.

Claims about uniquely human cognition inspired research on animal cooperation that challenged these claims. For example, it has been proposed that humans achieve high levels of cooperation because they have a unique sense of fairness (‘inequity aversion’) and thus split payoffs according to individual contributions [55]. A large body of research has shown that rudimentary forms of disadvantageous inequity aversion—aversion against receiving less than the interaction partner(s)—may be present in some non-human species (reviewed in [56]; but see [57]). In contrast, evidence for advantageous inequity aversion—aversion against receiving more than the interaction partner(s)—is currently lacking in non-human species and is apparently not even ubiquitous in humans [58].

In contrast to cognition, endocrinological research offers straightforward opportunities for a comparative approach, as humans are just standard mammals when it comes to hormones, neurohormones or neurotransmitters. Nevertheless, social scientists have often taken the lead in exploring the effect of these substances on helping behaviour. Research on the effects of oxytocin provides a case example. Oxytocin facilitates bonding between mammalian mothers and their offspring [59]. Research on humans revealed that this function may have been co-opted for creating bonds between unrelated individuals: increased oxytocin increases trust, without increasing risky behaviour overall, and increases within-group cooperation and between-group competition [60]. Oxytocin also mediates helping between unrelated individuals in several non-human mammals, including chimpanzees [61], dogs [62], vampire bats [63] and voles [64]. We note however that the robustness of various findings—in particular those based on exogenous application of oxytocin—is strongly debated (see [65]), and the jury is still out on how central-nervous and peripheral oxytocin mediates social behaviour in humans and other species. These concerns notwithstanding, research on endocrinological mechanisms underpinning social behaviour has been and will remain an interdisciplinary project.

## 4. Towards a more ecologically valid approach to helping in humans and other species

We have repeatedly emphasized how influential research on human helping was for biological research on other species. We believe that there is great potential for ever closer exchange of ideas and methods. Most importantly, biology has a long history of thinking about the problem of laboratory artefacts. While this does not mean that all biological research on helping is ecologically relevant [66], we will now highlight various important concerns about research on human helping from an ecological perspective. We postulate that further progress will depend on empirical data informing models rather than on experiments fitted to the assumptions of models.

First, we note that most experimental research taking an evolutionary approach to understand human cooperation precludes key features of the human cognitive toolbox, such as language, shared intentionality and shared group identity. This is because empiricists typically develop experiments in accordance with evolutionary game theory, which focuses on strategies rather than underlying mechanisms [67]. For instance, as theoretical models do not incorporate communication, subjects are typically prevented from talking to each other in experiments. Also, high levels of cooperation in humans typically occur between friends, colleagues or culturally created in-groups, while experiments often follow model assumptions and hence involve anonymous interactions between strangers. Thus, many experimental studies on humans are designed to test the predictions of general evolutionary game theory models rather than designed to explain how humans achieve extremely high levels of cooperation. As a consequence, we propose that typical economic experiments only yield baseline levels of human cooperation and that such levels may also be observed in various other species [68]. Of course, cooperation could similarly decrease under different conditions. A recent study [69] found that human cooperation increased under conditions in which subjects could talk to each other, in particular when in-group identity was triggered. We expect that the larger the group size and/or the incentive to cheat and/or the challenge to coordinate, the more important the human cognitive toolbox becomes to achieve high levels of cooperation. Studies that have allowed for communication during experiments have shown that communication can increase cooperation, either via gossip to deter cheating [70] or by allowing subjects to more efficiently coordinate actions [71]. Varying relationship quality between subjects will also be likely to yield additional insights, both in humans and other species.

An important goal for a biological approach to cooperation is to determine how the findings of abstract laboratory experiments apply in the real world [24,51,65,66]. Economic games that are typically used to study human behaviour are theory-driven but highly artificial. These abstract games can allow us to identify with a high degree of control how the various pillars that structure social interactions (e.g. anonymity, punishment, partner choice) directionally affect behaviour under the assumption that *ceteris paribus* these general effects apply in all settings [72]. Simple abstract games also allow a method for studying and quantifying variation in helping behaviour within and across populations (e.g. [73–75]). It is also likely that excluding more human-specific features like language has encouraged the interdisciplinary dialogue as both biologists and social scientists could use similar paradigms. Nevertheless, more effort should now be aimed at identifying if and how the findings from laboratory studies translate into real-world behaviours. Failing to do so runs the risk that empirical findings serve only to test the predictions of game-theoretical models and have little real-world relevance. We illustrate this point by summarizing discussion first on the meaning of payoff matrices in general, and then on indirect reciprocity as one specific example.

### (a) Payoff matrices

It is unclear to what extent the payoffs used in standard laboratory games approximate the payoffs of interactions that occur in the real world. The assumption that payoffs correlate positively with individual fitness holds in populations that are well mixed both with respect to genetic structure and with respect to potential interaction partners. The situation changes when limited migration and overlapping generations lead to kin structure and the potential for biological altruism, and when populations are structured into demes (groups) that compete with each other through contest or scramble competition. In those cases, material payoffs often provide a poor correlate of fitness. Instead, interacting individuals might become interdependent [2,3,76,77]. Interdependence has been proposed to be key to the evolution of extreme cooperation in humans [78]. Importantly, letting two highly interdependent individuals play a one-shot game with a prisoner's dilemma payoff matrix leads to confusion because the players' best option with respect to fitness is to either fully cooperate or to cooperate at least with some probability [24]. This is because interdependency can alter the fitness consequences of a prisoner's dilemma payoff matrix in such a way that fitness can be described by a prisoner's delight game (where cooperating yields higher payoffs/fitness via by-product benefits to the partner) or by a snowdrift game (where cooperating is under negative frequency dependent selection; figure 1). For example, zebra finches, a species with obligate bi-parental care, fail to cooperate with strangers in an experiment that uses an iterated prisoner's dilemma payoff matrix, but they show rather unconditional cooperation when paired with their social partner [79], perhaps due to interdependence between social partners [31]. A major question arising from the interdependence hypothesis to explain human uniqueness in levels of cooperation [78] is hence whether human interdependence is (or was) much more pronounced than in any other species, or whether some unique cognitive tools allowed humans to create extreme mutually beneficial interdependencies between unrelated individuals.

### (b) Indirect reciprocity

Indirect reciprocity also offers a cautionary tale on the importance of ecological validity. Indirect reciprocity occurs when an investment to help a recipient yields return benefits by an investment of a third party rather than by the initial recipient. Typically, indirect reciprocity therefore involves the existence of a reputation or an image score, and assessment rules determine how different actions affect reputation. A first detailed analysis of stable decision rules was provided by Kandori [19] and extended by Ohtsuki & Iwasa [80]. However, there is mixed evidence regarding whether people actually use these stable rules to judge the actions of others. Early evidence indicated that these second-order judgement rules were too cognitively complex to be used [81], while more recent evidence has indicated that reputation assessments can be predicated on second-order information regarding the context of helpful [82] or punitive [83] behaviour. Perhaps a more fundamental concern with the importance of indirect reciprocity as a general mechanism for supporting cooperation is the lack of real-world evidence that people behave in this way (but see [84]). One key paper that claims to have demonstrated indirect reciprocity in the real world [85] instead simply demonstrates that individuals show concern for reputation, which is not the same thing, as the crucial component—individuals with good reputation receive voluntary rewards from others—is missing.

One other major mechanism by which concern for reputation could yield downstream benefits is via partner choice. There is ample real-world evidence—including from non-human species—that partner choice is an important force underpinning cooperation, and the pressure to be chosen as a partner can lead to strategic [86] (and even competitive [29,87]) investments in reputation. Laboratory studies demonstrating indirect reciprocity may therefore be tapping into psychological mechanisms aimed at striking up mutually cooperative relationships with partners that have a good reputation, even though this is not possible in most laboratory studies of indirect reciprocity. Under the logic of error management [88], one could further predict that the high payoffs of striking up just one mutually productive relationship by ‘rewarding’ a helpful individual could sustain several small investments in rewards that do not ultimately lead to a relationship (cf. [89]). Error-management strategies could therefore result in behaviours that had the appearance of ‘rewarding’ helpful individuals in one-shot encounters, but would actually function to establish productive relationships. Experiments investigating the adaptive significance of acquiring a good reputation under real-world settings are now crucial to determine the relative importance of indirect reciprocity and reputation-based partner choice as mechanisms supporting cooperation.

This discussion highlights a larger issue of experimenter demand [90] in laboratory studies of human behaviour—changes in behaviour that occur because of what the subject believes to be appropriate in that context, rather than due to intrinsic motives or preferences. Most laboratory studies of indirect reciprocity have limited the behavioural options available to players. Thus, although indirect reciprocity is observed in laboratory experiments, we cannot rule out that these behaviours result from the expression of emotions whose only possible outlet in the context of the experiment is to reward helpful others. These emotions might well produce alternative behaviours in real-world scenarios that are nevertheless prevented by the rather impoverished selection available in the laboratory. Attempts to approximate reality by giving players more options in empirical games can affect the expression of behaviour (e.g. [36,83]). We suggest that the next wave of human evolutionary behavioural sciences ought to fully embrace these complexities in order to understand how behaviour in artificial laboratory settings relates to that in the real world.

## 5. General conclusion and outlook

We certainly support the idea that studies on human helping behaviour are relevant for biological research. In the tradition of Darwin [91], the highest relevance is achieved by studies that take an explicit evolutionary approach and refer at least to some extent to empirical and/or theoretical studies on other species. This view is also reflected in papers published recently in *Proceedings of the Royal Society B* (see electronic supplementary material).

Potentially, a unifying framework could be developed by studying how individuals decide whether to help, to cheat, to punish or to switch partners. This issue of decision-making links function and mechanisms. There is a clear need to study these processes [92] because humans and other animals do not use the simple strategies investigated in game-theoretic models (e.g. [93–95]). To determine why not, we should study social cognition—the mechanisms by which animals acquire, process, store and act on information from other individuals [96]—in its broadest biological sense. Perception of relevant stimuli can fundamentally affect decision-making. For example, it has been proposed that humans and other animals use heuristics or rules of thumb [97] to reach decisions quickly by ignoring a portion of the available information [98]. These processes are probably routed in well-established universal learning mechanisms, such as learning based on positive or negative reinforcement [99]. Excitingly, even in humans, reinforcement learning may explain various deviations as well as conformity with payoff-maximising behaviour [100]: for example, if behavioural option A yields a small gain in most trials, positive reinforcement may cause subjects to prefer this option over a more profitable option B that yields a high reward in few trials.

Recent theoretical studies have started to explicitly model reinforcement learning over the lifetime of individuals and selection on specific reinforcement learning parameters (i.e. the change in the probability of repeating a behaviour after receiving a reward) to study the consequences on social behaviour [101,102]. The models show that selection acting on reinforcement learning can yield cooperative solutions in an iterated prisoner's dilemma as well as consistent co-operators and defectors within pairs playing a repeated snowdrift game. What is still missing in the models is an integration of perceptual aspects. Early ethologists pointed out that learning needs to be studied within evolutionary history (i.e. within the ecology of a species). This is because evolution may shape the perception of species such that certain stimuli are more likely than others to elicit learning through positive or negative reinforcement. To give a concrete example, cleaner wrasse need to give priority to visitor clients over resident clients, as the former would otherwise swim off and visit another cleaner [34]. Species can be identified by their colour patterns and body shape, while the food (various species of ectoparasites) is highly overlapping between residents and visitors. As a consequence, cleaners can readily learn to preferentially approach an ephemeral food plate that differs from a permanent food plate only with respect to colour and patterns, a task that is extremely difficult for primates as well as rats and pigeons [103–105]. However, if the food items are coloured differently, or if food is hidden under cups of different colours, capuchin monkeys readily learn to prefer the ephemeral food source [106]. Taken together, the studies show that performance in the same biological market task varies according to a species's ability to perceive the relevant stimulus. Perception, strength of perceived reinforcement on actions and memory capacities (declarative, episodic or simply emotional) will all contribute to variation in cooperation within and between species.

In conclusion, we affirm that humans are just another species to test evolutionary theory. Research on human cooperation that takes a clear ecological or evolutionary perspective is as biologically relevant as research on any other species. Although helping has long been considered as an evolutionary puzzle that needs to reconciled with evolutionary theory and its emphasis on egoism, we believe that this puzzle has already been solved in the sense that there are many concepts that provide conditions under which biological altruism and cooperation can be favoured by selection. What is currently lacking is a general framework that can explain variation in helping tendencies within and between species, with human cooperation being the single most idiosyncratic data point. The current puzzle is thus why human cooperation is so unique on a quantitative level—and, moreover, why we also observe such striking variation in cooperation among different human individuals, groups and societies. We have argued that to solve the puzzle, we need to be more explicit about the links between cooperation and ecology and between cooperation and cognition (see also [107]). Both issues warrant a comparative approach, making research on human cooperation an interdisciplinary project of high biological relevance.

## Supplementary Material

Bshary-Raihani-humans as model system

## Data accessibility

This article has no data.

## References

[1] Maynard-Smith J, Szathmary E 1997 The major transitions in evolution. Oxford, UK: Oxford University Press.

[2] Hamilton W 1964 The genetical evolution of social behaviour. I. J. Theor. Biol. 7, 1–16. (10.1016/0022-5193(64)90038-4) 5875341

[3] Hamilton W 1964 The genetical evolution of social behaviour. II. J. Theor. Biol. 7, 17–52. (10.1016/0022-5193(64)90039-6) 5875340

[4] Neumann JV, Morgenstern O 1944 Theory of games and economic behavior. Princeton, NJ: Princeton University Press.

[5] Binmore K 2007 Playing for real: a text on game theory. Oxford, UK: Oxford University Press.

[6] Maynard-Smith J 1982 Evolution and the theory of games. Cambridge, UK: Cambridge University Press.

[7] Sigmund K, Nowak M 1999 Evolutionary game theory. Curr. Biol. 9, R503–R505. (10.1016/S0960-9822(99)80321-2) 10576907

[8] West SA, Griffin AS, Gardner A 2007 Social semantics: altruism, cooperation, mutualism, strong reciprocity and group selection. J. Evol. Biol. 20, 415–432. (10.1111/j.1420-9101.2006.01258.x) 17305808

[9] Lehmann L, Keller L 2006 The evolution of cooperation and altruism—a general framework and a classification of models. J. Evol. Biol. 19, 1365–1376. (10.1111/j.1420-9101.2006.01119.x) 16910958

[10] Bronstein JL 1994 Our current understanding of mutualism. Q. Rev. Biol. 69, 31–51. (10.1086/418432)

[11] Aumann R, Shapley L 1976 Long-term competition: a game theoretic analysis Mimeo.

[12] Trivers R 1971 The evolution of reciprocal altruism. Q. Rev. Biol. 46, 35–37. (10.1086/406755)

[13] Axelrod R, Hamilton W 1981 The evolution of cooperation. Science 211, 1390–1396. (10.1126/science.7466396) 7466396

[14] Smith A 1776 An inquiry into the nature and causes of the wealth of nations. London, UK: George Routledge and Sons.

[15] Noë R, Hammerstein P 1995 Biological markets. Trends Ecol. Evol. 10, 336–339. (10.1016/S0169-5347(00)89123-5) 21237061

[16] Hammerstein P, Noë R 2016 Biological trade and markets. Phil. Trans. R. Soc. B 371, 20150101 (10.1098/rstb.2015.0101) 26729940 PMC4760201

[17] McGregor PK 1993 Signalling in territorial systems: a context for individual identification, ranging and eavesdropping. Phil. Trans. R. Soc. Lond. B 340, 237–244. (10.1098/rstb.1993.0063)

[18] Alexander RD 1987 The biology of moral systems. New York, NY: Aldine de Gruyter.

[19] Kandori M 1992 The use of information in repeated games with imperfect monitoring. Rev. Econ. Stud. 59, 581–593. (10.2307/2297865)

[20] Boyd R, Gintis HM, Bowles S, Richerson PJ 2003 The evolution of altruistic punishment. Proc. Natl Acad. Sci. USA 100, 3531–3535. (10.1073/pnas.0630443100) 12631700 PMC152327

[21] Lehmann L, Rousset F, Roze D, Keller L 2007 Strong reciprocity or strong ferocity? A population genetic view of the evolution of altruistic punishment. Am. Nat. 170, 21–36. (10.1086/518568) 17853989

[22] West SA, Gardner A, Shuker DM, Reynolds T, Burton-Chellow M, Sykes EM, Guinnee MA, Griffin AS 2006 Cooperation and the scale of competition in humans. Curr. Biol. 16, 1103–1106. (10.1016/j.cub.2006.03.069) 16753564

[23] Hammerstein P 2003 Genetic and cultural evolution of cooperation. Cambridge, MA: MIT Press.

[24] Bshary R, Zuberbühler K, van Schaik CP 2016 Why mutual helping in most natural systems is neither conflict-free nor based on maximal conflict. Phil. Trans. R. Soc. B 371, 20150091 (10.1098/rstb.2015.0091) 26729931 PMC4760193

[25] Scott-Phillips TC, Dickins TE, West SA 2011 Evolutionary theory and the ultimate–proximate distinction in the human behavioral sciences. Perspect. Psychol. Sci. 6, 38–47. (10.1177/1745691610393528) 26162114

[26] Duguid S, Wyman E, Bullinger AF, Herfurth-Majstorovic K, Tomasello M 2014 Coordination strategies of chimpanzees and human children in a stag-hunt game*.* Proc. R. Soc. B 281, 20141973 (10.1098/rspb.2014.1973) PMC4213656 25320165

[27] Bshary R, Bronstein JL 2011 A general scheme to predict partner control mechanisms in pairwise cooperative interactions between unrelated individuals. Ethology 117, 271–283. (10.1111/j.1439-0310.2011.01882.x

[28] Fehr E, Gächter S 2002 Altruistic punishment in humans. Nature 415, 137–140. (10.1038/415137a) 11805825

[29] Wedekind C, Milinski M 2000 Cooperation through image scoring in humans. Science 288, 850–852. (10.1126/science.288.5467.850) 10797005

[30] Barclay P, Willer R 2007 Partner choice creates competitive altruism in humans. Proc. R. Soc. B 274, 749–753. (10.1098/rspb.2006.0209) PMC2197220 17255001

[31] Plott CR 1982 Industrial organization theory and experimental economics. J. Econ. Lit. 20, 1485–1527.

[32] Raihani NJ, Bshary R 2011 Resolving the iterated prisoner's dilemma: theory and reality. J. Evol. Biol. 24, 1628–1639. (10.1111/j.1420-9101.2011.02307.x) 21599777

[33] Côté IM 2000 Evolution and ecology of cleaning symbioses in the sea. Ocean. Mar. Biol. 38, 311–355.

[34] Bshary R 2010 Cooperation between unrelated individuals—a game theoretic approach. *In* Animal behaviour: evolution and mechanisms (ed. Kappeler P), pp. 213–240. Berlin, Germany: Springer.

[35] Raihani NJ, Grutter AS, Bshary R 2010 Punishers benefit from third-party punishment in fish. Science 327, 171 (10.1126/science.1183068) 20056883

[36] Raihani NJ, Pinto AI, Grutter AS, Wismer S, Bshary R 2012 Male cleaner wrasses adjust punishment of female partners according to the stakes. Proc. R. Soc. B 279, 365–370. (10.1098/rspb.2011.0690) PMC3223671 21676980

[37] Barclay P, Raihani NJ 2016 Partner choice versus punishment in human prisoner's dilemmas. Evol. Hum. Behav. 37, 263–271. (10.1016/j.evolhumbehav.2015.12.004)

[38] Bone JE, Wallace B, Bshary R, Raihani NJ, Mesoudi A 2016 Power asymmetries and punishment in a prisoner's dilemma with variable cooperative investment. PLoS ONE 11, e0155773 (10.1371/journal.pone.0155773) 27191958 PMC4871419

[39] Diekmann A 1985 Volunteer's dilemma. J. Conf. Res. 29, 605–610. (10.1177/0022002785029004003)

[40] Archetti M, Scheuring I 2012 Game theory of public goods in one-shot social dilemmas without assortment. J. Theor. Biol. 299, 9–20. (10.1016/j.jtbi.2011.06.018) 21723299

[41] Sefton M, Shupp R, Walker JM 2007 The effect of rewards and sanctions in provision if public goods. Econ. Inq. 45, 671–690. (10.1111/j.1465-7295.2007.00051.x)

[42] Arseneau-Robar TJM, Taucher AL, Müller E, van Schaik C, Bshary R, Willems EP 2016 Female monkeys use both the carrot and the stick to promote male participation in intergroup fights. Proc. R. Soc. B 283, 20161817 (10.1098/rspb.2016.1817) PMC5136586 27881752

[43] Bshary A, Bshary R 2010 Self-serving punishment of a common enemy creates a public good in reef fishes. Curr. Biol. 20, 2032–2035. (10.1016/j.cub.2010.10.027) 21055944

[44] Herbert-Read JE et al. 2016 Proto-cooperation: group hunting sailfish improve hunting success by alternating attacks on grouping prey. Proc. R. Soc. B 283, 20161671 (10.1098/rspb.2016.1671) PMC5124094 27807269

[45] Packer C, Ruttan L 1988 The evolution of cooperative hunting. Am. Nat. 132, 159–198. (10.1086/284844)

[46] Boesch C, Boesch H 1989 Hunting behavior of wild chimpanzees in the Tai National Park. Am. J. Phys. Anthropol. 78, 547–573. (10.1002/ajpa.1330780410) 2540662

[47] Mitani JC, Watts DP 2001 Why do chimpanzees hunt and share meat? Anim. Behav. 61, 915–924. (10.1006/anbe.2000.1681)

[48] Hofer H, East M 1995 Population dynamics, population size, and the commuting system of Serengeti spotted hyenas. In Serengeti II: dynamics, management, and conservation of an ecosystem, vol. 2 (eds Sinclair ARE, Arcese P), p. 332 Chicago, IL: University of Chicago Press.

[49] Packer C, Pusey A 1985 Asymmetric contests in social mammals: respect, manipulation and age-specific aspects. In Evolution: essays in honour of John Maynard Smith (eds Greenwood PJ, Slatkin M), pp. 173–186. Cambridge, UK: Cambridge University Press.

[50] Burton-Chellew MN, May RM, West SA 2013 Combined inequality in wealth and risk leads to disaster in the climate change game. Clim. Change 120, 815–830. (10.1007/s10584-013-0856-7)

[51] West SA, Griffin AS, Gardner A, Diggle SP 2006 Social evolution theory for microorganisms. Nat. Rev. Microbiol. 4, 597–607. (10.1038/nrmicro1461) 16845430

[52] Raihani NJ, Bshary R 2015 Why humans might help strangers. Front. Behav. Neurosci. 9, 2531 (10.3389/fnbeh.2015.00039 PMC4335183 25750619

[53] MacLean EL 2016 Unraveling the evolution of uniquely human cognition. Proc. Natl Acad. Sci. USA 113, 6348–6354. (10.1073/pnas.1521270113) 27274041 PMC4988573

[54] Csibra G, Gergely G 2009 Natural pedagogy. Trends Cogn. Sci. 13, 148–153. (10.1016/j.tics.2009.01.005) 19285912

[55] Fehr E, Schmidt KM 1999 A theory of fairness, competition and cooperation. Quart. J. Econ. 114, 817–868. (10.1162/003355399556151)

[56] Brosnan SF, de Waal FB 2014 Evolution of responses to (un)fairness. Science 346, 1251776 (10.1126/science.1251776) 25324394 PMC4451566

[57] Bräuer J, Hanus D 2012 Fairness in non-human primates? Soc. Just. Res. 25, 256–276. (10.1007/s11211-012-0159-6)

[58] Blake PR et al 2015 The ontogeny of fairness in seven societies. Nature 528, 258–261. (10.1038/nature15703 26580018

[59] Donaldson ZR, Young LJ 2008 Oxytocin, vasopressin, and the neurogenetics of sociality. Science 322, 900–904. (10.1126/science.1158668) 18988842

[60] De Dreu CK 2012 Oxytocin modulates cooperation within and competition between groups: an integrative review and research agenda. Horm. Behav. 61, 419–428. (10.1016/j.yhbeh.2011.12.009) 22227278

[61] Crockford C, Wittig RM, Langergraber K, Ziegler TE, Zuberbühler K, Deschner T 2013 Urinary oxytocin and social bonding in related and unrelated wild chimpanzees. Proc. R. Soc. B 280, 20122765 (10.1098/rspb.2012.2765) PMC3574389 23345575

[62] Romero T, Nagasawa M, Mogi K, Hasegawa T, Kikusui T 2014 Oxytocin promotes social bonding in dogs. Proc. Natl Acad. Sci. USA 111, 9085–9090. (10.1073/pnas.1322868111) 24927552 PMC4078815

[63] Carter GG, Wilkinson GS 2015 Intranasal oxytocin increases social grooming and food sharing in the common vampire bat *Desmodus rotundus*. Horm. Behav. 75, 150–153. (10.1016/j.yhbeh.2015.10.006) 26475061

[64] Burkett JP, Andari E, Johnson Z, Curry DC, de Waal FB, Young LJ 2016 Oxytocin-dependent consolation behavior in rodents. Science 351, 375–378. (10.1126/science.aac4785) 26798013 PMC4737486

[65] Nave G, Camerer C, McCullough ME 2015 Does oxytocin increase trust in humans? A critical review of research. Perspect. Psychol. Sci. 10, 772–789. (10.1177/1745691615600138) 26581735

[66] Noë R 2006 Cooperation experiments: coordination through communication versus acting apart together. Anim. Behav. 71, 1–18. (10.1016/j.anbehav.2005.03.037)

[67] McAuliffe WH, McCullough ME 2014 Validation is a Galilean enterprise. Evol. Hum. Behav. 38, 279–280. (10.1016/j.evolhumbehav.2016.11.003)

[68] Taborsky M, Frommen JG, Riehl C 2016 Correlated pay-offs are key to cooperation. Phil. Trans. R. Soc. B 37, 20150084 (10.1098/rstb.2015.0084) PMC4760186 26729924

[69] McClung JS, Placi S, Bangerter A, Clément F, Bshary R In press The language of cooperation: shared intentionality drives variation in helping as a function of group membership. Proc. R. Soc. B10.1098/rspb.2017.1682 PMC5627217 28931743

[70] Sommerfeld RD, Krambeck H-J, Semmann D, Milinski M 2007 Gossip as an alternative for direct observation in games of indirect reciprocity. Proc. Natl Acad. Sci. USA 104, 17 435–17 440. (10.1073/pnas.0704598104) PMC2077274 17947384

[71] Brosnan SF, Parrish A, Beran MJ, Flemming T, Heimbauer L, Talbot CF, Lambeth SP, Schapiro SJ, Wilson BJ. 2011 Responses to the assurance game in monkeys, apes, and humans using equivalent procedures. Proc. Natl Acad. Sci. USA 108, 3442–3447. (10.1073/pnas.1016269108) 21300874 PMC3044417

[72] Camerer C 2011 The promise and success of lab–field generalizability in experimental economics: a critical reply to Levitt and List. SSRN Electron. J. (10.2139/ssrn.1977749)

[73] Henrich J et al. 2005 ‘Economic man’ in cross-cultural perspective: behavioral experiments in 15 small-scale societies. Behav. Brain Sci. 28, 795–855. 16372952 10.1017/S0140525X05000142

[74] Herrmann B, Thöni C, Gächter S 2008 Antisocial punishment across societies. Science 319, 1362–1367. (10.1126/science.1153808) 18323447

[75] Lamba S, Mace R 2011 Demography and ecology drive variation in cooperation across human populations. Proc. Natl. Acad. Sci. USA 108, 14 426–14 430. (10.1073/pnas.1105186108) PMC3167540 21831836

[76] Eshel I, Shaked A 2001 Partnership. J. Theor. Biol. 208, 457–474. (10.1006/jtbi.2000.2232) 11222050

[77] Roberts G 2005 Cooperation through interdependence. Anim. Behav. 70, 901–908. (10.1016/j.anbehav.2005.02.006)

[78] Tomasello M, Melis AP, Tennie C, Wyman E, Herrmann E 2012 Two key steps in the evolution of human cooperation. Curr. Anthropol. 53, 673–692. (10.1086/668207)

[79] St-Pierre A, Larose K, Dubois F 2009 Long-term social bonds promote cooperation in the iterated prisoner's dilemma. Proc. R. Soc. B 276, 4223–4228. (10.1098/rspb.2009.1156) PMC2821343 19740884

[80] Ohtsuki H, Iwasa Y 2007 Global analyses of evolutionary dynamics and exhaustive search for social norms that maintain cooperation by reputation. J. Theor. Biol. 244, 518–531. (10.1016/j.jtbi.2006.08.018) 17030041

[81] Milinski M, Semmann D, Bakker TCM, Krambeck H-J 2001 Cooperation through indirect reciprocity: image scoring or standing strategy? Proc. R. Soc. Lond. B 268, 2495–2501. (10.1098/rspb.2001.1809) PMC1088906 11747570

[82] Swakman V, Molleman L, Ule A, Egas M 2016 Reputation-based cooperation: empirical evidence for behavioral strategies. Evol. Hum. Behav. 37, 230–235. (10.1016/j.evolhumbehav.2015.12.001)

[83] Raihani NJ, Bshary R 2015 Third-party punishers are rewarded, but third-party helpers even more so. Evolution 69, 993–1003. (10.1111/evo.12637) 25756463

[84] Khadjavi M 2016 Indirect reciprocity and charitable giving—evidence from a field experiment. Manag. Sci. (10.1287/mnsc.2016.2519)

[85] Yoeli E, Hoffman M, Rand DG, Nowak MA 2013 Powering up with indirect reciprocity in a large-scale field experiment. Proc. Natl Acad. Sci. USA 110, 10 424–10 429. 10.1073/pnas.1301210110 PMC3690615 23754399

[86] Pinto AI, Oates J, Grutter AS, Bshary R 2011 Cleaner wrasses *Labroides dimidiatus* are more cooperative in the presence of an audience. Curr. Biol. 21, 1140–1144. (10.1016/j.cub.2011.05.021) 21700458

[87] Raihani NJ, Smith S 2015 Competitive helping in online giving. Curr. Biol. 25, 1183–1186. (10.1016/j.cub.2015.02.042) 25891407

[88] Johnson DDP, Blumstein DT, Fowler JH, Haselton MG 2013 The evolution of error: error management, cognitive constraints, and adaptive decision-making biases. Trends Ecol. Evol. 28, 474–481. (10.1016/j.tree.2013.05.014) 23787087

[89] Delton AW, Krasnow M, Cosmides L, Tooby J 2011 Evolution of direct reciprocity under uncertainty can explain human generosity in one-shot encounters. Proc. Natl. Acad. Sci. USA 108, 13 335–13 340. (10.1073/pnas.1102131108) PMC3156224 21788489

[90] Zizzo DJ 2010 Experimenter demand effects in economic experiments. Exp. Econ. 13, 75–98. (10.1007/s10683-009-9230-z)

[91] Darwin C 1871 The descent of man, and selection in relation to sex. London, UK: John Murray.

[92] Hammerstein P, Stevens JR 2012 Evolution and the mechanisms of decision making. Cambridge, MA: MIT Press.

[93] De Waal FB 2000 Attitudinal reciprocity in food sharing among brown capuchin monkeys. Anim. Behav. 60, 253–261. (10.1006/anbe.2000.1471) 10973728

[94] Fehr E, Fischbacher U 2003 The nature of human altruism. Nature 425, 785–791. (10.1038/nature02043) 14574401

[95] Kummerli R, Burton-Chellew MN, Ross-Gillespie A, West SA 2010 Resistance to extreme strategies, rather than prosocial preferences, can explain human cooperation in public goods games. Proc. Natl Acad. Sci. USA 107, 10 125–10 130. (10.1073/pnas.1000829107) PMC2890432 20479253

[96] Shettleworth SJ 2010 Cognition, evolution, and behavior, 2nd edn Oxford, UK: Oxford University Press.

[97] Gigerenzer G, Todd PM, ABC Research Group. 1999 Simple heuristics that make us smart. Oxford, UK: Oxford University Press.

[98] Hutchinson JM, Gigerenzer G 2005 Simple heuristics and rules of thumb: where psychologists and behavioural biologists might meet. Behav. Proc. 69, 97–124. (10.1016/j.beproc.2005.02.019) 15845293

[99] Kacelnik A 2012 Putting mechanisms into decision-making. In Evolution and the mechanisms of decision making (eds Hammerstein P, Stevens JR), pp. 21–38. Cambridge, MA: MIT Press.

[100] Erev I, Roth AE 2014 Maximization, learning, and economic behavior. Proc. Natl Acad. Sci. USA 111, 10 818–10 825. (10.1073/pnas.1402846111) PMC4113920 25024182

[101] Dridi S, Lehmann L 2014 On learning dynamics underlying the evolution of learning rules. Theor. Popul. Biol. 91, 20–36. (10.1016/j.tpb.2013.09.003) 24055617

[102] Dridi S, Lehmann L 2015 A model for the evolution of reinforcement learning in fluctuating games. Anim. Behav. 104, 87–114. (10.1016/j.anbehav.2015.01.037)

[103] Salwiczek LH et al. 2012 Adult cleaner wrasse outperform capuchin monkeys, chimpanzees and orang-utans in a complex foraging task derived from cleaner–client reef fish cooperation. PLoS ONE 7, e49068 (10.1371/journal.pone.0049068) 23185293 PMC3504063

[104] Zentall TR, Case JP, Berry JR 2017 Rats' acquisition of the ephemeral reward task. Anim. Cogn. 20, 419–425. (10.1007/s10071-016-1065-3) 27988824

[105] Zentall TR, Case JP, Luong J 2016 Pigeon's (*Columba livia*) paradoxical preference for the suboptimal alternative in a complex foraging task. J. Comp. Psychol. 130, 138–144. (10.1037/com0000026) 27064201

[106] Prétôt L, Bshary R, Brosnan SF 2016 Factors influencing the different performance of fish and primates on a dichotomous choice task. Anim. Behav. 119, 189–199. (10.1016/j.anbehav.2016.06.023)

[107] Brosnan SF, Bshary R 2010 Cooperation and deception: from evolution to mechanisms. Phil. Trans. R. Soc. B 365, 2593–2598. (10.1098/rstb.2010.0155) 20679104 PMC2942876

