# Supplementary material for: Helping in humans and other animals: a fruitful interdisciplinary dialogue
Source: Proc Biol Sci. 2017 Sep 27;284(1863):20170929. doi: 10.1098/rspb.2017.0929 (PMC5627196; doi:10.1098/rspb.2017.0929)
Supplement: Bshary-Raihani-humans as model system [file rspb20170929supp1.docx]

Supporting information

We were curious what kind of recent papers on human cooperation are published in a biological journal like Proceedings of the Royal Society B. Searching on the PRSB website, we found twelve papers on human helping published since 2015. All use an evolutionary framework [1-12]. Five of these papers provide evolutionary game theory models on decision-making [1], morality based on partner choice [2], indirect reciprocity [3] and public goods games [4,5]. The empirical papers also cover a diverse range of topics, i.e. decision-making [6-8], life history decisions in the context of kin selection [9], effects of oxytocin [10], morality [11] and religion [12]. Only the latter two papers and one modelling paper [5] do not refer to any literature on other species.

References

1. Bear A, Kagan A, Rand DG. 2017 Co-evolution of cooperation and cognition: the impact of imperfect deliberation and context-sensitive intuition. *Proc. R. Soc. B* **284**: 20162326.
2. Debove S, André JB, Baumard N. 2015 Partner choice creates fairness in humans. *Proc. R. Soc. B* **282**, 20150392.
3. Tanaka H, Ohtsuki H, Ohtsubo Y. 2016 The price of being seen to be just: an intention signalling strategy for indirect reciprocity. *Proc. R. Soc. B* **283**, 20160694.
4. Szolnoki A, Perc M. 2015 Antisocial pool rewarding does not deter public cooperation. *Proc. R. Soc. B* **282**, 20151975.
5. Schlüter M, Tavoni, A, Levin S. 2016 Robustness of norm-driven cooperation in the commons. *Proc. R. Soc. B* 283, 20152431.
6. Burton-Chellew MN, Nax HH, West SA. 2015 Payoff-based learning explains the decline in cooperation in public goods games. *Proc. R. Soc. B* **282**, 20142678.
7. Capraro V, Cococcioni G. 2015 Social setting, intuition and experience in laboratory experiments interact to shape cooperative decision-making. *Proc. R. Soc. B* **282**, 20150237.
8. Burton-Chellew MN, El Mouden C, West SA. 2017 Social learning and the demise of costly cooperation in humans. *Proc. R. Soc. B* **284**, 20170067.
9. Hooper PL, Gurven M, Winking J, Kaplan HS. 2015 Inclusive fitness and differential productivity across the life course determine intergenerational transfers in a small-scale human society. *Proc. R. Soc. B* **282**, 20142808.
10. Kret ME, De Dreu CK. 2017 Pupil-mimicry conditions trust in partners: moderation by oxytocin and group membership. *Proc. R. Soc. B* **284**, 20162554.
11. Fessler DM, Barrett HC, Kanovsky M, Stich S, Holbrook C, Henrich J, ... & Pisor AC. 2015 Moral parochialism and contextual contingency across seven societies. *Proc. R. Soc. B* **282**, 20150907.
12. Watts J, Greenhill SJ, Atkinson QD, Currie TE, Bulbulia, J, Gray RD. 2015 Broad supernatural punishment but not moralizing high gods precede the evolution of political complexity in Austronesia. *Proc. R. Soc. B* **282**, 20142556.
